# The adjunctive role of metformin in patients with mild to moderate ulcerative colitis: a randomized controlled study

**DOI:** 10.3389/fphar.2025.1507009

**Published:** 2025-03-19

**Authors:** Ammena Y. Binsaleh, Sahar M. El-Haggar, Sahar K. Hegazy, Maha M. Maher, Monir M. Bahgat, Thanaa A. Elmasry, Sarah Alrubia, Amsha S. Alsegiani, Mamdouh Eldesoqui, Mostafa M. Bahaa

**Affiliations:** ^1^ Department of Pharmacy Practice, College of Pharmacy, Princess Nourah bint Abdulrahman University, Riyadh, Saudi Arabia; ^2^ Clinical Pharmacy Department, Faculty of Pharmacy, Tanta University, El-Gharbia Government, Tanta, Egypt; ^3^ Pharmacy Practice Department, Faculty of Pharmacy, Horus University, New Damietta, Egypt; ^4^ Internal Medicine Department, Faculty of Medicine, Mansoura University, Mansoura, Egypt; ^5^ Internal Medicine Department, Faculty of Medicine, Horus University, New Damietta, Egypt; ^6^ Pharmacology and Toxicology Department, Faculty of Pharmacy, Tanta University, Tanta, Al-Gharbia, Egypt; ^7^ Pharmacology and Toxicology Department, Faculty of Pharmacy, Sinai University, Arish campus, Egypt; ^8^ Pharmaceutical Chemistry Department, College of Pharmacy, King Saud University, Riyadh, Saudi Arabia; ^9^ Department of Basic Medical Sciences, College of Medicine, AlMaarefa University, Riyadh, Saudi Arabia

**Keywords:** ulcerative colitis, disease activity index, metformin, calprotectin, nitric oxide

## Abstract

**Background:**

Metformin, hypoglycemic medication, is recognized for its diverse properties and its capacity to influence the inflammatory pathways. Medications with anti-inflammatory and anti-oxidative characteristics have been demonstrated to be able to elicit and sustain remission in ulcerative colitis (UC), chronic inflammatory disorder of the bowel. Studies in both preclinical and clinical settings have looked into the several metabolic pathways via which metformin protects against UC.

**Aim:**

To assess efficacy of metformin as adjunctive therapy in patients with mild to moderate UC.

**Methods:**

This clinical research was double-blinded, randomized, controlled, and involved 60 patients with mild to moderate UC. The participants were randomly assigned to one of two groups (n = 30). The control group was given 1 g of mesalamine three times a day (t.i.d.) for a period of 6 months (mesalamine group). The metformin group was given 500 mg of metformin twice daily and 1 g of mesalamine t. i.d. For a period of 6 months. Patients with UC were assessed by a gastroenterologist using the disease activity index (DAI) both at the beginning of treatment and 6 months thereafter. To evaluate the drug’s biological efficacy, measurements of fecal calprotectin, serum C-reactive protein (CRP), interleukin 10 (IL-10), and nitric oxide (NO) were taken both before and after treatment.

**Study outcomes:**

Decrease in DAI and change in the level of measured serum and fecal markers.

**Results:**

The metformin group displayed a statistical reduction in DAI (*p* = 0.0001), serum CRP (*p* = 0.019), NO (*p* = 0.04), and fecal calprotectin (*p* = 0.027), as well as a significant increase in IL-10 (*p* = 0.04) when compared to the mesalamine group. There was a significant direct correlation between DAI and calprotectin (p < 0.0001, r = 0.551), and between DAI and CRP (p < 0.0001, r = 0.794). There was a significant negative correlation between DAI and IL-10 (p = 0.0003, r = 0.371).

**Conclusion:**

Metformin may be an effective adjunct drug in management of patients with mild to moderate UC by decreasing DAI and other inflammatory markers that were involved in the pathogenesis of UC.

**Clinical Trial Registration:**

identifier NCT05553704.

## 1 Introduction

Crohn’s disease (CD) and ulcerative colitis (UC) are two of the many gastrointestinal tract disorders that are included in the category of inflammatory bowel diseases (IBD), which are long-term inflammatory diseases. More than 0.3% of persons in North America, Australia, and Europe have IBD, making it a very prevalent condition in western countries. This significantly burdens the medical care industry (Wanchaitanawong et al., 2022). IBD is rare (0.001%–0.05%) in Asia, Africa, and South America, but a recent study indicated that its incidence has increased yearly by 4%–15% in these newly modernized regions, raising concerns about the disease’s potential impact on global health (Gajendran et al., 2019).

The pathogenesis of UC is complex and involves an impaired intestinal barrier, external factors, genetic susceptibility, bacterial imbalance in the gut, and an inappropriate immunological response (Guan 2019). The course of treatment for UC is determined by the severity and course of the disease. Currently, patients with mild to moderate cases can receive conventional treatments like amino salicylates (ASA), immunomodulators, and corticosteroids, or biologic agents like anti-interleukin (IL) 12/23, anti-tumor necrosis factor (TNF), anti-Janus kinase (JAK), and anti-integrin (Silaghi et al., 2022). Regardless of the abundance of biologic medications on the market, therapeutic outcomes are not always desirable. Patients considered to be primary non-responders to anti-TNFs ranged from 10 to 40 percent (Marsal et al., 2022). A progressive absence of response also occurred in 10–50 percent of patients (Marsal et al. 2022). Thus, between 20 and 30 percent of patients with UC and 30 to 40 percent of those with CD required intestinal surgery at some point in their lives. In addition, IBD patients find it challenging to take several currently prescribed drugs due to their negative effects (Burisch et al., 2022). Clinicians have therefore been searching for novel therapies or strategies to repurpose drugs that are successful in treating UC.

Cytokines play a major role in the inflammatory processes that promote inflammation and the pathophysiology of UC by, among other things, producing inflammatory mediators and activating inflammatory pathways. In UC, they directly result in tissue destruction and mucosal inflammation, which set off condition-specific immune reactions (Neurath 2024). Numerous inflammatory mediators were involved in the pathogenesis of UC like interleukins, chemokines, and other mediators (Neurath 2024).

Currently, the most fully investigated inflammatory indicators are CRP and fecal calprotectin (Yoon et al., 2014). Despite the documented association between endoscopic activity and CRP, the evidence are currently insufficient to justify its widespread application in UC (Magro et al., 2016). There are several positive findings for fecal calprotectin, which demonstrate extremely strong association with clinical response, endoscopic parameters, as well as mucosal restoration in UC (Angriman et al., 2007; Nakov et al., 2019; Yoon et al. 2014).

Reactive oxygen and reactive nitrogen species (ROS and RNS) have long been implicated in both the causes and development of UC (Muro et al., 2024). The injured lamina propria of patients with UC showed considerable neutrophil infiltration and a rise in myeloperoxidase levels, which were similar to the epithelia. In mice, inducible nitric oxide synthase (iNOS) gene deletion was demonstrated to drastically reduce the development and severity of colitis (McCafferty et al., 2000; Piechota-Polanczyk and Fichna 2014). In UC, iNOS is thought to be responsible for significantly elevated NO generation in the mucosa and in areas of inflammation in conjunction with nitrotyrosine (Tachibana et al., 2020). iNOS-derived NO enhances cytokine production in large intestine, leading to neutrophil infiltration, for example, by stimulating the production of intracellular adhesion molecule (ICAM) and P-selectin, resulting in colonic tissue injury (Piechota-Polanczyk and Fichna 2014).

Metformin, with its comparatively low cost as well as favorable safety characteristics, is the initial therapy for patients with type 2 diabetes (McCreight et al., 2016). Beyond its anti-diabetic effects, previous studies have highlighted its potential therapeutic benefits, including anticancer activity, cardiovascular protection, anti-aging effects, and anti-inflammatory properties (Anisimov 2013; Leone et al., 2014; Rena and Lang 2018). Notably, metformin has been shown to enhance goblet cell numbers in the gastrointestinal tract, suggesting a mucus-protective role (Xue et al., 2016). Additionally, multiple preclinical studies have demonstrated that metformin reduces colitis severity by inhibiting key inflammatory pathways, including p38 mitogen-activated protein kinase (MAPK), Jun N-terminal kinases (JNK), phosphorylated signal transducer and activator of transcription 3 (pSTAT3), and nuclear factor kappa-light-chain-enhancer of activated B cells (NF-κB) (Deng et al., 2018; Di Fusco et al., 2018; El-Haggar et al., 2024). EL-mahdy et al., reported that metformin reduced disease activity index (DAI) and inflammatory markers in oxazolone induced colitis (El-Mahdy et al., 2021). Clinically, metformin alleviated inflammation, decreased serum inflammatory markers, and upregulated tight junction proteins in patients with mild to moderate UC (El-Haggar et al., 2024). Metformin is associated with improved IBD outcomes in patients with Type 2 diabetes mellitus in propensity-matched cohort study (Petrov et al., 2024).

Despite these promising findings, clinical evidence supporting the use of metformin as an adjuvant therapy in UC remains limited. To the best of our knowledge, this study is among the first randomized controlled trials to evaluate the efficacy of metformin in patients with mild to moderate UC. In light of these investigations, our study aims to provide novel insights into the potential therapeutic role of metformin in UC management by assessing its effects on DAI, fecal calprotectin, and key inflammatory biomarkers such as serum CRP, NO, and IL-10.

## 2 Patients and methods

This study was a part of our previously published work about repurposing of metformin in patients with mild to moderate UC (El-Haggar et al., 2024). Between November 2022 and December 2023, sixty patients who satisfied the eligibility criteria were selected from the Gastroenterology Department. The ethical review committee of the Mansoura University Faculty of Medicine authorized this research. The Helsinki Declaration and its 1964 revisions were followed and adhered in the study’s design and methods. The patients were told that they might withdraw from the research at any time. Both patients and physicians were kept blinded about the kind of exposure and randomization. An unblinded chemist administered study medications to participants to guarantee appropriate treatment assignment; the chemist was not involved in the assessment of research results.

### 2.1 Inclusion criteria

Patients above the age of eighteen, both male and female, were enrolled in this study. Effective contraception and a negative pregnancy test should be provided to female patients. This clinical study only covered mild to moderate patients of UC.

### 2.2 Exclusion criteria

Patients receiving systemic or rectal steroids, immunosuppressive medications, or having severed type UC were excluded. Additionally excluded were patients with renal or hepatic impairment in order to avoid the adverse metabolic consequences of metformin. Diabetic patients were excluded to specifically assess the anti-inflammatory and immunomodulatory effects of metformin in UC without the confounding influence of its glucose-lowering properties. Individuals have a history of lactic acidosis, total or partial colectomy, and colorectal malignancy were also ineligible. Lastly, women who were nursing and those who were receiving metformin treatment for polycystic ovarian syndrome either before or now were not included.

### 2.3 Study design

The safety and effectiveness of metformin as an adjuvant medication to mesalamine in treatment of UC were assessed in this clinical study.

Under the NCT05553704 code, this study was listed at www.Clinical.Trials.gov.in.2022.

**FIGURE 1:**
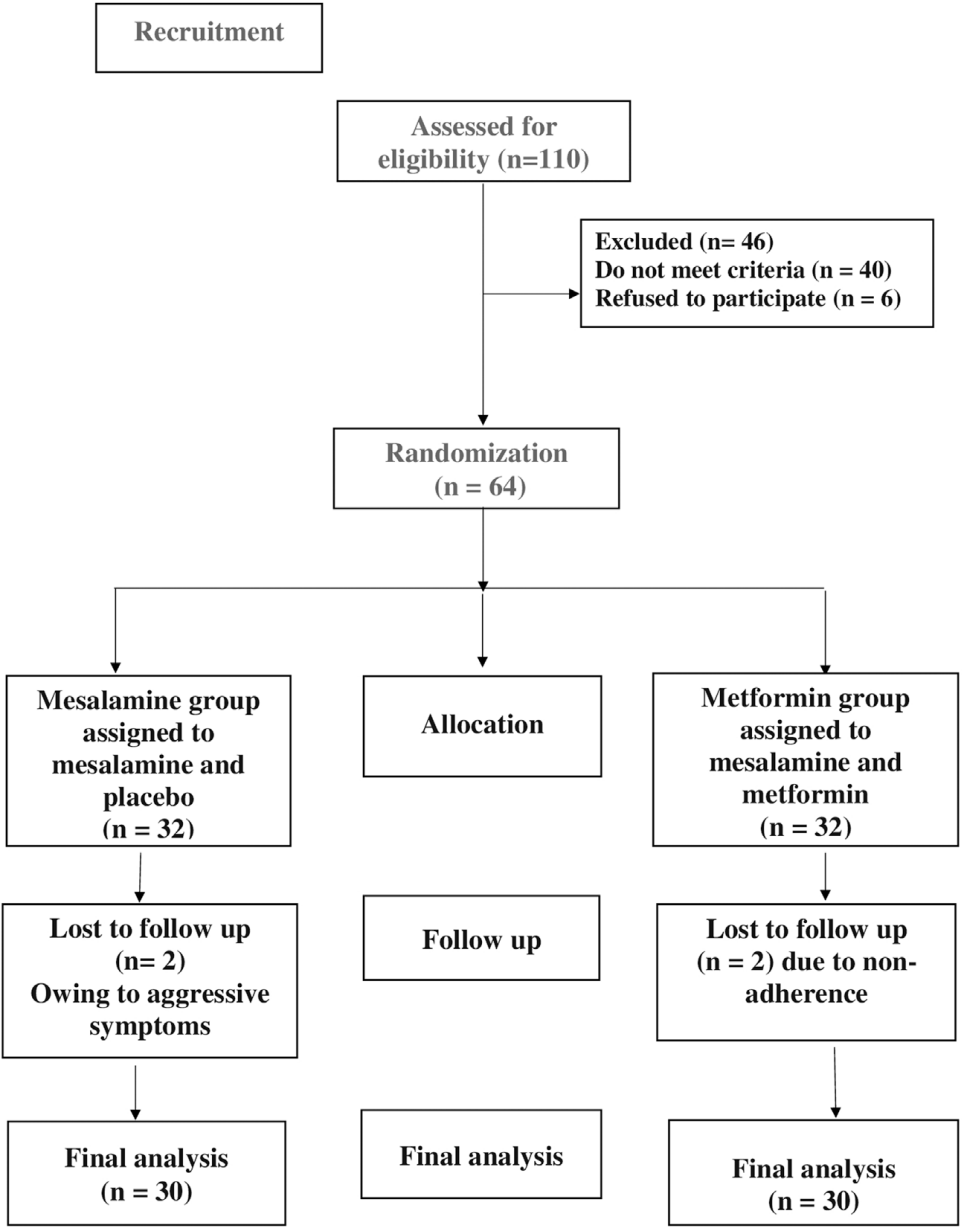
CONSORT diagram showing the flow of patients during study.

The patients were split into two groups at random (n = 30), as Figure 1 CONSORT flow diagram illustrates. A computer random number generator was used to select randomly permuted blocks for the randomization process. Thirty patients were randomly allocated to one of two groups after fulfilling the eligibility requirements and giving their written, informed consent.

Group 1 (mesalamine group): Patients in this group received 1 g of mesalamine tablets t. i.d. (PentasaR 500 mg, Multi Pharm, Egypt) and a placebo for a period of 6 months.
Group 2 (metformin group): Patients in this group received 500 mg metformin tablets bid (GlucophageR 500 mg, Mina Pharm, Egypt) and 1 g of mesalamine tablets t. i.d. (PentasaR 500 mg, Multi Pharm, Egypt) for a period of 6 months.

### 2.4 Sample size calculations

The sample size for our study was determined based on recommendations by Sima and Lewis (Sim and Lewis 2012) who suggest a minimum of 22 participants per group for detecting small to medium effect sizes in pilot studies. Since there were not any studies that investigated the effect of metformin on DAI in patients with mild to moderate UC, this study was designed to be a pilot one. Given that our study serves as a preliminary investigation into the efficacy of metformin as an adjuvant therapy in UC, we aimed to ensure adequate statistical power while maintaining feasibility. To enhance the robustness of our findings, we increased the sample size to 32 patients per group, accounting for a power of 0.80, an α-error of 0.05 (two-tailed), and a 20% dropout rate. This approach aligns with standard recommendations for pilot studies, ensuring that our study provides meaningful preliminary data to inform future larger-scale trials.

### 2.5 Study protocol

In alongside the enrollment checks, UC patients received thorough mental, physical, and psychological assessments. Patients were randomly assigned to receive either a placebo tablet and 1 g of mesalamine tablets given t. i.d. (mesalamine group) or 1 g of mesalamine tablets taken t. i.d. Together with 500 mg of metformin tablets orally administered bid (metformin group), in accordance with the CONSORT guidelines. Zeta Pharma Company produced placebo tablets, which were identical in appearance to metformin tablets. Along with nutritional and lifestyle counseling, all medications were administered orally to the patients. Based on earlier research, the doses of metformin and mesalamine that have been chosen are 500 mg bid (Garber et al., 1997) and 1 g t. i.d. (Kruis et al., 2003), respectively. The chosen dosage of 500 mg twice a day falls within the widely used therapeutic range, and prior research examining its non-glycemic effects has demonstrated that it is well tolerated in non-diabetic people. Metformin by itself is not (Holstein and Egberts 2003; Lalau et al., 1998) or infrequently (Group UPDS, 1998) linked to hypoglycemia, which is characterized by symptoms and indicators of hypoglycemia and/or plasma glucose levels below 3.3 mmol/l as well as a clinical reaction to glucose delivery. The reported risks of hypoglycemia for metformin users ranged from 0% to 21%, in a previous review (Bolen et al., 2007). Metformin may have a lesser risk of hypoglycemia than other oral antidiabetic medications because it does not directly boost insulin production. However, hypoglycemia in patients using metformin may occur in association with strenuous physical activity or fasting (Bodmer et al., 2008). Furthermore, we advised all patients to take metformin after meals, this will result in reducing the incidence of hypoglycemia. Importantly, we carefully monitored all participants for any signs of hypoglycemia or adverse effects throughout the study to ensure patient safety.

### 2.6 Follow-up

Monthly phone calls and meetings were used to follow up with patients. At the first visit, patients received a full medical history, liver function [alanine amino-transferase (ALT) and aspartate amino-transferase (AST)] and kidney function testing (serum creatinine (SrCr), and complete blood counts in order to exclude out any organic abnormalities. Serum biomarkers (NO, CRP, IL-10) and fecal calprotectin were measured.

### 2.7 Study outcomes

Change in DAI and measured serum and fecal markers (IL-10, NO, CRP, calprotectin).

### 2.8 Evaluation of colitis

In accordance with Mitsuru Seo et al., the Disease Activity Index (DAI) was computed for every patient both before and after 6 months of treatment (Seo et al., 1992).

DAI = 13 x bowel movements +60 x blood stool +0.5 x ESR - 4 x HB - 15 x albumin +200. Index values below 150, values between 150 and 220, and values above 220 nearly corresponded to mild, moderate, and severe disease, respectively.

Bowel movements: Reflecting the frequency of diarrhea as an indicator of disease severity.

Blood in stool: A critical marker of mucosal inflammation and ulceration.

Erythrocyte Sedimentation Rate (ESR): A systemic inflammatory marker.

Hemoglobin (HB): Representing the impact of chronic inflammation and potential blood loss.

Serum albumin: A marker of nutritional status and disease severity.

Constant factor (200): Used for standardization of the scale.

### 2.9 Therapeutic assessments

Therapeutic assessment was done by measuring DAI, serum markers (NO, CRP, IL-10), and fecal calprotectin.

### 2.10 Sample collection

Before the investigation began and 6 months after the treatment, 10 mL of blood were drawn from the antecubital vein. The blood sample was then centrifuged for 10 mins at 4,500 g (Hettich Zentrifugen EBA 20) after the blood was progressively transferred into test tubes and allowed to coagulate. Two serum aliquots were taken; the first was used for routine tests on the kidney and liver, and the second one was frozen at −80°C to measure specific cytokine levels.

Stools that had been weighed were dissolved in regular saline and centrifuged. Clear supernatants were used to analyze the calprotectin in fecal material.

### 2.11 Biochemical analysis

Using commercially available enzyme-linked immunosorbent assay (ELISA) kits, the serum levels of IL-10 (catalogue no.: 201-12-0,103), NO (catalogue no.: 201–12–1,511), CRP (catalogue no.: DY1707), and fecal calprotectin (catalogue no.: 201-12-5,461) were measured in accordance with manufacturer’s guidelines. With the exception of the CRP kits, which came from R&D Systems China Co., Ltd., the kits were supplied by Sunredio, Shanghai, China.

The human IL-10 level in samples was measured using a double-antibody ELISA kit. IL-10 was added to a monoclonal antibody enzyme well that had been pre-coated with human IL-10 monoclonal antibody for incubation; then IL-10 antibodies labelled with biotin were added and combined with Streptavidin-HRP to form an immune complex; finally, incubation and washing to remove the uncombined enzyme were carried out. Then chromogen solution A and B were added, changing the color of the liquid from blue to yellow as a result of the acid effect. All markers were measured in the same manner.

### 2.12 Statistical analysis

The statistical analysis was performed using Prism version 9 from GraphPadsoftware, Inc., San Diego, California, United States. Using the Shapiro-Wilk method, the normality of a continuous variable was examined. Using the Wilcoxon test and the Student’s t-test before and after therapy, significant variations were observed within the group for nonparametric and parametric data, respectively. To find any statistically significant variations between groups before and after therapy, the Man Whitney test and the unpaired Student’s t-test were used for nonparametric and parametric data, respectively. While the mean ± SD was utilized to convey quantitative data, numbers, median, and the interquartile range, were utilized to represent qualitative markers. The Pearson correlation test was used to find the correlation between the normally distributed parameters. The Chi-square test and Fisher exact test were utilized for categorical results. There were two tails to every p-value, with less than 0.05 p-value being regarded as statistically significant.

## 3 Results

### 3.1 Clinical and demographic characteristics

The current study found no statistically significant differences between mesalamine and metformin groups in terms of baseline characteristics, including platelet count (p = 0.147), SrCr (p = 0.617), age (p = 0.113), sex (p = 0.796), weight (p = 0.726), height (p = 0.404), ALT (p = 0.289), AST (p = 0.467), and glycated hemoglobin (p = 0.643). Regarding the baseline demographic data which are the same as our previously published one (El-Haggar et al., 2024), we put this table in the Supplementary Table S1. Due to non-compliance with medication, two patients were lost to follow-up in the metformin group. Two other patients were dropped from the mesalamine group and transferred to immunosuppressive combination therapy. Since sixty patients finished the trial, all measurable parameters' statistical analyses were carried out in accordance with protocol.

### 3.2 Effect of study medications on measured markers

At the start of the study, there were no significant differences in all measured markers between the two study groups (p > 0.05). (Table 1).

**TABLE 1:** Effect of study medications on measured parameters.

	Mesalamine group (n = 30)			Metformin group (n = 30)			P value
Character	Before treatment	After treatment	P value	Before treatment	After treatment	P value	After treatment
DAI	179.4 ± 26.37	110.6 ± 33.4	<0.0001*	170.9 ± 31.22	81.47 ± 20.51	<0.0001*	0.0001**
IL-10 (pg/mL)	170 ± 17.16	178.9 ± 13.76	0.039*	172.1 ± 12.04	187.7 ± 18.46	0.0007*	0.04**
NO (μmol/L)	240 (153.5–255.5)	187.7 (125.3–267.3)	0.007#	230.8 (137.5–256)	146 (100.5–194.2)	0.0003#	0.04##
CRP (pg/mL)	152.3 ± 9.521	65.66 ± 8.407	<0.0001*	154.3 ± 7.964	59.80 ± 10.42	<0.0001*	0.019**
Calprotectin (ng/mL)	27.07 ± 3.822	23.49 ± 3.718	0.0005*	30.44 ± 4.543	21.13 ± 4.313	<0.0001*	0.027**

Data was displayed as median, interquartile range, and mean ± SD, mesalamine group; UC, patients treated with mesalamine and placebo, Metformin group; UC, patients treated with mesalamine plus metformin; DAI, disease activity index; IL-10, interleukin 10; NO, nitric oxide, CRP, C-reactive protein. (*) and (#) level of significance within the same group by paired t-test and Wilcoxon test, respectively. (**) and (##) level of significance between groups using unpaired t-test and Man Witney test, respectively. Significance at (p < 0.05).

After treatment, mesalamine group showed significant changes in all measured markers when compared to its baseline values as followed: DAI (179.4 ± 26.37 versus 110.6 ± 33.4, *p* < 0.0001), IL-10 (170 ± 17.16 versus 178.9 ± 13.76, *p* = 0.039), NO (240 (153.5–255.5) versus 187.7 (125.3–267.3), *p* = 0.007), CRP (152.3 ± 9.521 versus 65.66 ± 8.407, *p* < 0.0001), and calprotectin (27.07 ± 3.822 versus 23.49 ± 3.718, *p* = 0.0005) (Table 1).

Metformin group displayed significant changes in all measured markers when compared to its baseline values as followed: DAI (170.9 ± 31.22 versus 81.47 ± 20.51, *p* < 0.0001), IL-10 (172.1 ± 12.04 versus 187.7 ± 18.46, *p* = 0.0007), NO (230.8 (137.5–256) versus 146 (100.5–194.2), *p* = 0.0003), CRP (154.3 ± 7.964 versus 59.80 ± 10.42, *p* < 0.0001), and calprotectin (30.44 ± 4.543 versus 21.13 ± 4.313, *p* < 0.0001) (Table 1).

Between group comparisons, unpaired t-test revealed that there were significant changes in DAI (*p* = 0.0001), IL-10 (*p* = 0.04), CRP (*p* = 0.019), and calprotectin (*p* = 0.027). ManWitney test revealed that there was a significant change in NO (*p* = 0.04) level between the two groups.

### 3.3 Correlation analysis between the studied parameters

**FIGURE 2:**
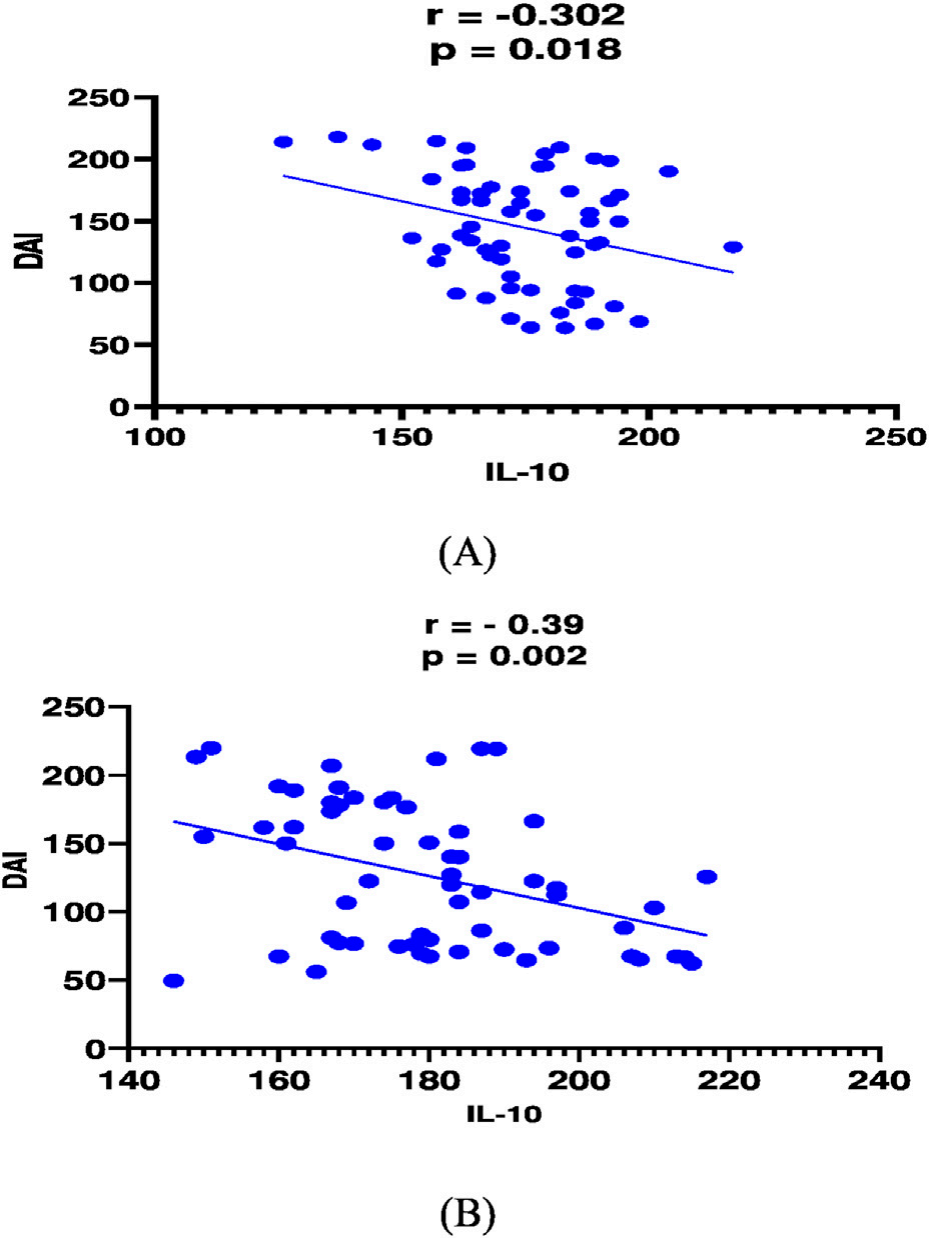
Correlation analysis between Disease activity index (DAI) and IL-10 in mesalamine group **(A)** and metformin group **(B)**.

**FIGURE 3:**
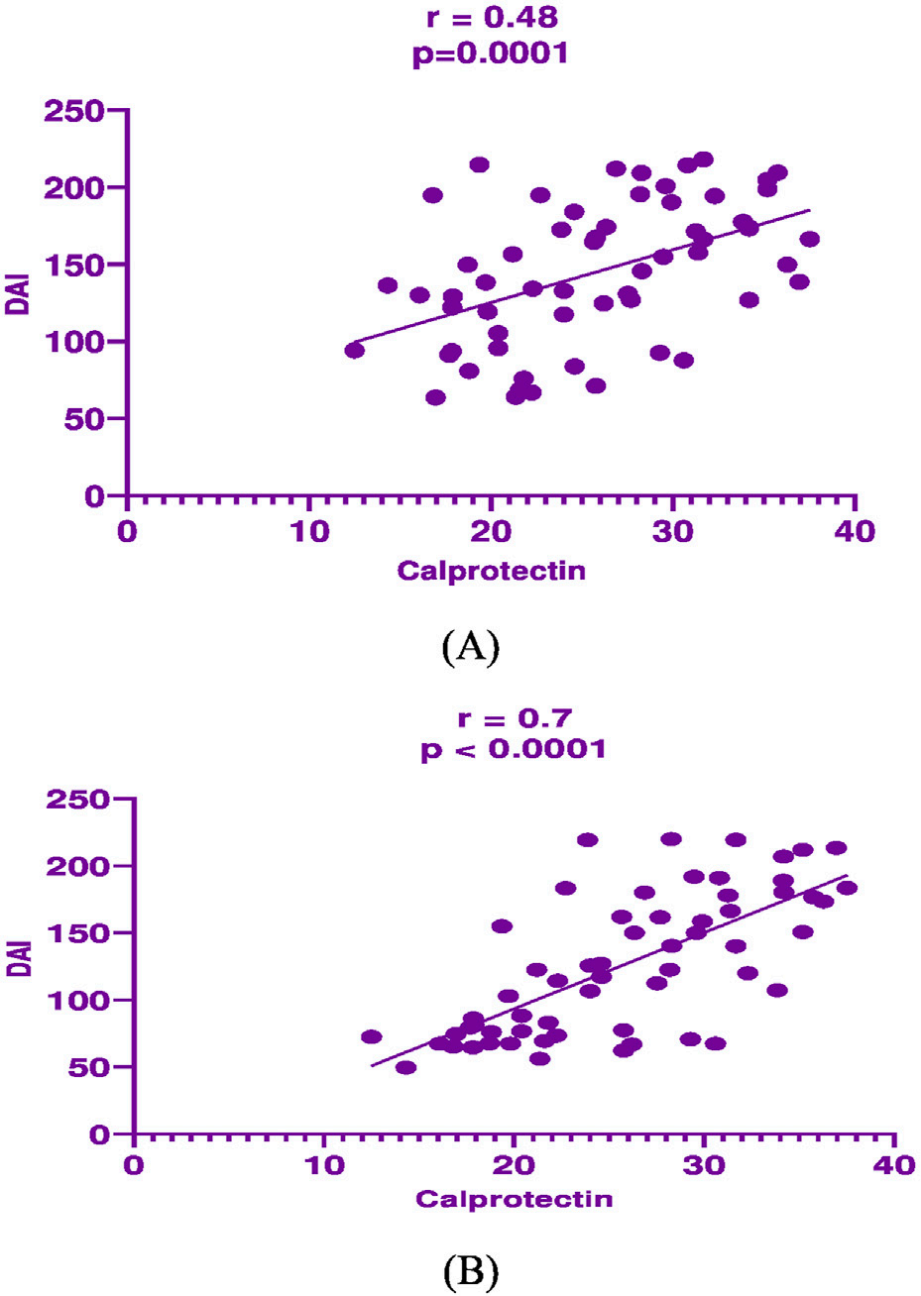
Correlation analysis between Disease activity index (DAI) and fecal calprotectin in mesalamine group **(A)** and metformin group **(B)**.

**FIGURE 4:**
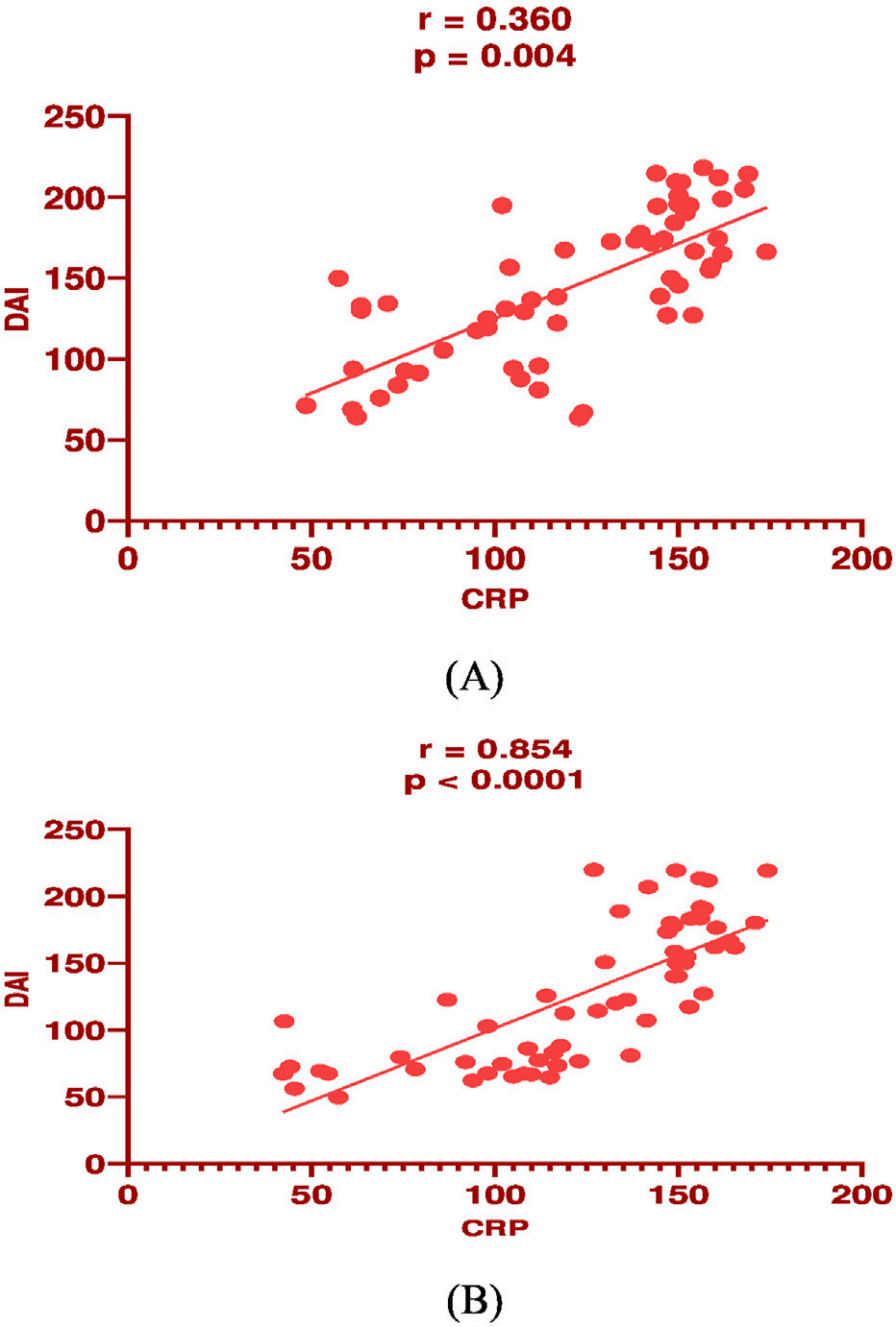
Correlation analysis between Disease activity index (DAI) and serum C-reactive protein (CRP) in mesalamine group **(A)** and metformin group **(B)**.

In mesalamine group, there was a significant direct correlation between DAI and CRP [(p = 0.004, r = 0.36), Figure 2A], and between DAI and calprotectin [(p = 0.0001, r = 0.48), Figure 3A] and a significant negative correlation between DAI and IL-10 [(p = 0.018, r - = 0.302), Figure 4A].

In metformin group, there was a significant direct correlation between DAI and CRP [(*p* < 0.0001, r = 0.854), Figure 2B], and between DAI and calprotectin [(*p* < 0.0001, r = 0.7), Figure 3B] and a significant negative correlation between DAI and IL-10 [(p = 0.002, r - = 0.39), Figure 4B].

## 4 Discussion

Ulcerative colitis (UC) is a form of inflammatory bowel disease characterized by persistent and recurring inflammation in the colon, leading to symptoms such as abdominal pain, bloody diarrhea, more frequent bowel movements, and other systemic effects. Its increasing incidence has been linked to lifestyle changes in developing countries, along with several other factors (Ke et al., 2021). Reactive oxygen species and a rise in inflammatory responses have long been known to have a role in the development of this illness, even though its exact cause is unknown (El-Mahdy et al. 2021). Elevated levels of oxidative stress indicators, pro-inflammatory cells, and mediators were observed in the colonic tissue of UC patients and together they contribute to the loss of integrity and ulceration of mucosa (Wanchaitanawong et al. 2022).

We have published part of this work previously, and this is a continuation of this previous study (El-Haggar et al., 2024). Several clinical (El-Haggar et al., 2024; Kabel et al., 2017; Petrov et al. 2024) and preclinical studies (El-Mahdy et al. 2021; Ke et al. 2021; Liu et al., 2021) were conducted and evaluated the repurposing of metformin in inflammatory bowel diseases. It is worthy to note that this is the first clinical study to evaluate the effect of metformin on patients with mild to moderate UC by decreasing NO, calprotectin, CRP, DAI, and increasing anti-inflammatory cytokine (IL-10).

Metformin administration in addition to mesalamine substantially lowered oxidative stress and decreased colitis more than mesalamine monotherapy. In light of these findings, the current study offers a crucial knowledge of the part metformin plays in the medication’s protective effects against oxidative stress and inflammation. The findings demonstrate that metformin lessens UC severity. There is consistent evidence that in mouse colitis, metformin has anti-inflammatory effects. Metformin decreased colonic inflammation by activating the AMP-activated protein kinase, according to research by Di Fusco et al., (Di Fusco et al. 2018). According to Lee et al., metformin reduced colitis by increasing the AMPK signaling cascade and inhibiting STAT3 activation (Lee et al., 2015). Additionally, Deng et al. demonstrated that metformin alleviated mice colitis by protecting against gastrointestinal barrier disruption through AMPKa1-dependent suppression of JNK pathway stimulation (Deng et al. 2018).

Metformin combination with mesalamine in the current study significantly reduced DAI when compared to mesalamine monotherapy. These results were in line with previous reports (Koh et al., 2014; Liu et al. 2021). Metformin (100 mg/kg and 500 mg/kg) taken orally considerably reduced the degree of severity of colitis as determined by body weight loss, DAI, and colon length (Koh et al. 2014). Dextran sulfate-(DSS)-induced acute colitis was significantly improved with metformin pretreatment (Koh et al. 2014). EL-mahdy et al., reported that metformin either alone or in combination with mesalamine significantly reduced macroscopic and microscopic scores in oxazolone induced colitis (El-Mahdy et al. 2021). All of these observations and previous findings highlighted the protective role of metformin in reducing mucosal damage in UC. When compared to the same parameters before therapy in a previous trial, metformin administration to patients with UC resulted in a significant drop in colonic endoscopic score as well as an alleviation of the histopathology (Kabel et al. 2017). Metformin reduced the symptoms of colitis brought on by the DSS, as seen by reduction in disease index, increased body weight, and improved mucosal integrity (Liu et al. 2021).

It was previously believed that oxidative stress was a major factor in the pathophysiology of UC. Persistent bowel inflammation in UC is usually attributed to an excess of ROS and RNS, respectively (Balmus et al., 2016). Oxidative stress is generated in UC when there is typically an imbalance between ROS and antioxidant capacity. Upon synthesis, ROS engage in molecular complex interactions to trigger oxidative damage within cells. This damage can impact lipids, proteins, and nucleic acids, resulting in the creation of lipid peroxides, disruption of enzyme functions, and split DNA strands (Jena et al., 2012). According to Ashabi et al.'s research, the nuclear factor erythroid 2 related factor (Nrf2) pathway is activated by metformin’s promotion of AMPK activation, which in turn causes its antioxidant and anti-inflammatory actions (Ashabi et al., 2015). This was consistent with the current study’s findings, which indicated that patients with UC had a markedly elevated NO level at the onset of treatment. Metformin treatment dramatically decreased NO in comparison to both the mesalamine group and its baseline value. These findings coincide with previous researches (El-Mahdy et al. 2021; Pandey et al., 2017; Sadeghi et al., 2019). Wang et al., evaluated how metformin inhibited iNOS, which in turn reduced the amount of NO produced by monocytes (Wang et al., 2020). The present investigation found that the administration of mesalamine considerably decreased the level of NO, and these findings are consistent with prior reports (He et al., 2019). Metformin also enhanced the ability of antioxidant enzymes by raising lower glutathione levels (Sadeghi et al., 2019). Strong antioxidant and free radical scavenger properties have been demonstrated in mesalamine (Kaiser et al., 1999).

Fecal calprotectin levels were significantly lower in the metformin group as compared to the baseline and mesalamine groups. These outcomes were consistent with earlier research (Boshra 2022; Djaja et al., 2019). When there is persistent inflammation in the gut, polymorphonuclear neutrophils move from the bloodstream to the gastrointestinal mucosa. Neutrophils flow into the lumen as a result of any inflammatory process-induced damage of the mucosal architecture, releasing calprotectin, which is subsequently expelled in stool (D'Amico et al., 2020). The quantity of calprotectin found in the feces is positively correlated with the intensity of UC (Grgić et al., 2022). In comparison with mesalamine alone, the current investigation showed that metformin plus mesalamine dramatically decreased calprotectin. Grip and Olof showed that in patients with Crohn’s disease (CD), there was a strong relationship between the proinflammatory mediators and the calprotectin quantity (Grip and Janciauskiene 2009). Fecal calprotectin levels in UC patients' stools are a good indicator of mucosal healing and are correlated with both histologic and endoscopic inflammation (Theede et al., 2015). In support with the current research, there was a strong positive correlation between DAI and fecal calprotectin. These results support the notion that calprotectin may be a prognostic and diagnostic non-invasive tool in UC. Taina Sipponen et al., reported that inflammatory bowel diseases activity was assessed by fecal calprotectin and lactoferrin and there was a strong correlation between Crohn’s disease activity index and endoscopic findings (Sipponen et al., 2008).

The current study demonstrated that combination therapy between metformin and mesalamine significantly reduced CRP when compared to mesalamine alone. These findings were in line with previous researches in the same field (Chen et al., 2017; Vermeire et al., 2004). IL-6, IL-1β, and TNF-α stimulate the production of CRP, a pentameric protein that is virtually entirely generated by hepatocytes. (Tall 2004). The primary acute-phase protein is CRP. CRP has a baseline value of 1 mg/L, and its levels are somewhat influenced by genetics. When there is an infection or inflammation in the acute phase, CRP levels rise sharply. When the inflammatory process is managed, CRP concentrations likewise drop rapidly (Vermeire et al. 2004). CRP levels significantly decreased after metformin treatment, especially in obese women (Chen et al. 2017). The effect of metformin on acute phase reactant proteins may be due to its potent anti-inflammatory activity by decreasing NF-kB, reducing insulin resistance, and activating AMPK pathways (Ye et al., 2018). The results of the current study revealed that there was a strong correlation between DAI and CRP level. These findings were matched with previous studies (Osada et al., 2008; Schoepfer et al., 2009). The clinical and endoscopic indices were used to prospectively analyze 134 UC patients in the Schoepfer et al. study. The highest correlation (r = 0.503) was seen between endoscopic disease activity and CRP. When CRP was raised, the overall accuracy of detecting endoscopically active illness was 62% (Schoepfer et al. 2009). In a different Japanese investigation, the total endoscopic and histological results were connected with the CRP concentration; specifically, the activity of proximal colonic lesions was positively correlated with both CRP and erythrocyte sedimentation rate (ESR) (Osada et al. 2008). These findings were validated by Henriksen et al., who demonstrated that CRP levels at diagnosis elevated as the disease’s severity increased in UC patients (Henriksen et al., 2008).

Several studies proved that metformin exhibited potent anti-inflammatory activity and boosting the levels of anti-inflammatory cytokines such as IL-10 (Jenkins et al., 2012; Sharma et al., 2013). These observations were matched with our study as there was a significant increase in serum IL-10 level by synergistic combination of metformin and mesalamine than mesalamine alone. The finding that mice lacking IL-10 and its receptor exhibit bowel inflammation on their own suggested a close relationship between IL-10 and gastrointestinal mucosal equilibrium. These factors have made IL-10’s anti-inflammatory qualities an extremely interesting target for IBD treatment (Shouval et al., 2014). Several studies established that mice deficient in IL-10 developed colitis and metformin treatment significantly boosts IL-10 (Berg et al., 2002; Elliott et al., 2004). However, a conflicting result showed that in a jejunal cell model of IBD, metformin increased IL-10 transcription while decreasing it in other cases (Wu et al., 2018). The shorter duration of the investigation, which was intended to mimic an acute inflammatory response, may have contributed to this contradictory results. This notion was strongly supported by previous research showing that metformin reduced the production of IL-10 in macrophages following acute lipopolysaccharide (LPS) exposure (Postler et al., 2021). IL-10 is a cytokine with anti-inflammatory properties that is also referred to as human cytokine synthesis inhibitory factor (CSIF). Monocytes are the main producers of this cytokine, with lymphocytes producing it to a lesser degree (Sharma et al. 2013). This mediator has multiple impacts on immunoregulation and inflammatory cascades. It suppresses the expression of costimulatory molecules, Th1 cytokines, and major histocompatibility complex (MHC) class II Ags on macrophages. Additionally, it improves B cell growth, survival, and production of antibodies (Sharma et al. 2013). This cytokine is involved in the control of the JAK-STAT signaling cascade and has the ability to suppress NF-jB activation (Sharma et al. 2013). Metformin limits the generation of IL-1β and increases IL-10 in lipopolysaccharide-stimulated macrophages via inhibiting the formation of ROS from nicotinamide adenine dinucleotide hydrogen (NADH) ubiquinone oxidoreductase (Kelly et al., 2015). It has been reported that metformin has a potent anti-inflammatory activity through peroxisome proliferator-activated receptors (PPAR) dependent mechanisms (Qu and Qu 2019).

Last but not at least, the mesalamine group displayed a significant reduction in DAI, serum NO, IL-10, CRP, and fecal calprotectin when compared to its baseline values. These findings were consistent with earlier publications. (Alarfaj et al., 2024; Bahaa et al., 2024; El-Haggar et al., 2024). Mesalamine has been widely utilized in the treatment of mild to moderate cases of UC, hence it is quite likely that mesalamine itself is responsible for these observations (Bahaa et al. 2024). These results align with previous studies that examined the impact of mesalamine on UC (El-Mahdy et al. 2021; El-Haggar et al., 2024). Mesalamine possesses anti-inflammatory and apoptotic properties and suppresses inflammatory cytokines through a PPAR-gamma-dependent manner (Rousseaux et al., 2005).

The study drugs were mostly accountable for the therapeutic outcomes because at the beginning of the investigation, there were not significant differences in the groups' clinical or demographic characteristics.

## 5 Conclusion

While preclinical studies have suggested that metformin may exert anti-inflammatory and immunomodulatory effects in IBD through AMP-activated protein kinase (AMPK) activation, inhibition of NF-κB signaling, and modulation of gut microbiota, clinical evidence remains scarce. Our study is the first clinical trial to investigate the effects of metformin as an adjunct to mesalamine in patients with UC, with a specific focus on fecal calprotectin, NO, CRP, and IL-10 as biomarkers of inflammation. Metformin may be a potential adjunctive treatment for patients with UC. This may be brought about by the combination of metformin and mesalamine’s synergistic anti-inflammatory and antioxidant properties, as well as their capacity to reduce inflammatory indicators and the DAI.

### 5.1 Merits of the study

1. Novelty and Clinical Relevance: Our trial is among the first to provide clinical evidence of metformin’s potential benefits in UC patients, specifically evaluating its effects on inflammatory biomarkers (CRP, IL-10, calprotectin), oxidative stress markers (NO), and disease activity indices.
2. Synergistic Mechanism with Mesalamine: The combination of metformin and mesalamine may enhance anti-inflammatory effects due to their complementary mechanisms of action. While mesalamine primarily inhibits prostaglandin synthesis and suppresses local inflammation, metformin reduces systemic inflammation and oxidative stress.
3. Potential Biomarker Utility: The study highlights fecal calprotectin, NO, CRP, and IL-10 as possible indicators of metformin’s therapeutic response in UC, paving the way for future biomarker-driven treatment strategies.
4. Clinical Implications for Drug Repurposing: Metformin is a well-characterized, widely available, and cost-effective drug with an established safety profile, making it an attractive candidate for adjunctive UC therapy.

### 5.2 Demerits and limitations of the study

1. Short Follow-up Duration: The relatively brief study period limits the ability to assess long-term effects of metformin on disease remission, relapse rates, and sustained biomarker changes.
2. Small Sample Size: The limited number of participants reduces statistical power and generalizability, necessitating larger, multicenter trials to confirm our findings.
3. Lack of Variable Dosage Analysis: The study utilized a fixed metformin dose, whereas dose-response variations could provide insights into the optimal therapeutic regimen for UC patients.
4. Absence of a Healthy Control Group and Metformin only Group: While including healthy participants and metformin only group for comparative analysis would have strengthened the findings, ethical restrictions in Egypt, particularly regarding invasive procedures such as colonic biopsies, posed challenges. IRB of Mansoura University rejected the recruitment of healthy control and metformin only groups.
5. Metformin-Related Adverse Effects Not Monitored: Given metformin’s known effects on glucose metabolism, vitamin B12 levels, and weight loss. Tracking serum glucose, body weight, and vitamin B12 levels would have been beneficial. These parameters should be considered before and after treatment in future studies.

Overall, despite these limitations, our study provides preliminary clinical evidence supporting metformin as a promising adjunct to mesalamine in UC management. Further randomized controlled trials with larger sample sizes, longer follow-up periods, and dose-response evaluations are warranted to validate these findings and explore metformin’s full therapeutic potential in UC.

## Data Availability

The raw data supporting the conclusions of this article will be made available by the authors, without undue reservation.
