# Supplementary material for: The adjunctive role of metformin in patients with mild to moderate ulcerative colitis: a randomized controlled study
Source: Front Pharmacol. 2025 Mar 19;16:1507009. doi: 10.3389/fphar.2025.1507009 (PMC11969268; doi:10.3389/fphar.2025.1507009)
Supplement: Supplementary file 1 [file Table1.docx]

**Table 1: Clinical, demographic and laboratory data of the patients.**

| Parameter | Mesalamine group | Metformin group | P value |
| --- | --- | --- | --- |
| Age (years) | 43.60 ± 11.31 | 48.03 ± 10.02 | 0.113 |
| Sex (M/F) | 15 /15 | 16 /14 | 0.796 |
| Weight (kg) | 72.23 ± 9.687 | 71.40 ± 8.677 | 0.726 |
| Height (m^2^) | 1.636 ± 0.122 | 1.662 ± 0.114 | 0.404 |
| HbA1C | 4.52 ± 0.419 | 4.57 ± 0.397 | 0.643 |
| Serum ALT (IU/L) | 43.20 ± 10.68 | 45.83 ± 8.247 | 0.289 |
| Serum AST (IU/L) | 39.13 ± 9.87 | 36.97 ± 12.88 | 0.467 |
| SrCr (mg/dL) | 0.794 ± 0.144 | 0.812 ± 0.137 | 0.617 |
| Platelet count (10^3^/mm3) | 194.8 ± 19.38 | 201.1 ± 13.14 | 0.147 |

Data was presented as mean ±SD and numbers, Mesalamine group, UC patients treated with mesalamine and placebo, Metformin group, UC patients treated with mesalamine plus metformin, M: Male, F: Female, HbA1C, glycated haemoglobin, ALT, alanine aminotransferase; AST, aspartate aminotransferase; SrCr, serum creatinine. Significance at (*p* < 0.05)**.**
